# Modeling Eastern Russian High Arctic Geese (*Anser fabalis, A. albifrons*) during moult and brood rearing in the ‘New Digital Arctic’

**DOI:** 10.1038/s41598-021-01595-7

**Published:** 2021-11-11

**Authors:** Diana Solovyeva, Inga Bysykatova-Harmey, Sergey L. Vartanyan, Alexander Kondratyev, Falk Huettmann

**Affiliations:** 1 Institute of Biological Problems of the North, Far East Branch, Russian Academy of Sciences, Magadan, Russia; 2 Institute of Biological Problems of the Cryolithozone, Siberian Branch, Russian Academy of Sciences, Yakutsk, Russia; 3 North-East Interdisciplinary Scientific Research Institute N. A. Shilo, Far East Branch, Russian Academy of Sciences, Magadan, Russia; 4 EWHALE lab – Institute of Arctic Biology, Biology & Wildlife Department, University of Alaska Fairbanks (UAF), Fairbanks, Alaska USA

**Keywords:** Biological techniques, Ecology, Zoology, Climate sciences, Ecology, Environmental sciences, Mathematics and computing

## Abstract

Many polar species and habitats are now affected by man-made global climate change and underlying infrastructure. These anthropogenic forces have resulted in clear implications and many significant changes in the arctic, leading to the emergence of new climate, habitats and other issues including digital online infrastructure representing a ‘New Artic’. Arctic grazers, like Eastern Russian migratory populations of Tundra Bean Goose *Anser fabalis* and Greater White-fronted Goose *A. albifrons,* are representative examples and they are affected along the entire flyway in East Asia, namely China, Japan and Korea. Here we present the best publicly-available long-term (24 years) digitized geographic information system (GIS) data for the breeding study area (East Yakutia and Chukotka) and its habitats with ISO-compliant metadata. Further, we used seven publicly available compiled Open Access GIS predictor layers to predict the distribution for these two species within the tundra habitats. Using BIG DATA we are able to improve on the ecological niche prediction inference for both species by focusing for the first time specifically on biological relevant population cohorts: post-breeding moulting non-breeders, as well as post-breeding parent birds with broods. To assure inference with certainty, we assessed it with 4 lines of evidence including alternative best-available open access field data from GBIF.org as well as occurrence data compiled from the literature. Despite incomplete data, we found a good model accuracy in support of our evidence for a robust inference of the species distributions. Our predictions indicate a strong publicly best-available relative index of occurrence (RIO). These results are based on the quantified ecological niche showing more realistic gradual occurrence patterns but which are not fully in agreement with the current strictly applied parsimonious flyway and species delineations. While our predictions are to be improved further, e.g. when synergetic data are made freely available, here we offer within data caveats the first open access model platform for fine-tuning and future predictions for this otherwise poorly represented region in times of a rapid changing industrialized ‘New Arctic’ with global repercussions.

## Introduction

**Figure 1:**
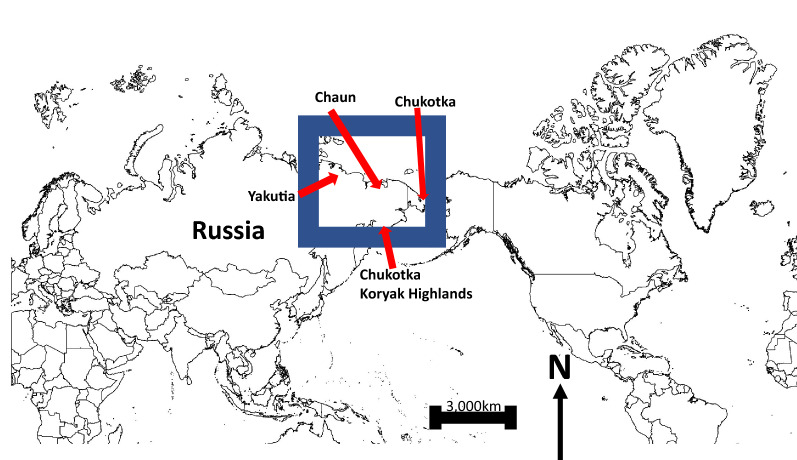
Circumpolar arctic and study area with general survey areas.

Adding to the natural global climate processes the human-caused global warming as well as new technology and industrialization also result into major shifts and dire ecological consequences giving rise to a ’New Arctic’^1,2^. The Russian Eastern Arctic—Yakutia and Chukotka—are part of this process and are also core zones supporting dense breeding populations of waterbirds in the circumpolar Arctic (Fig. 1). Waterbird populations in general, and geese and swans populations in particular, of Arctic Yakutia and Chukotka have been studied for a rather long time^3–10^. However, they are still comparatively poorly represented in the western literature thus far^11–17^ show overall recent geese population estimates and outlines of their distribution in North East Asia; however this is based on expert opinion rather than on distribution models based on transparent data^18^ for a review of expertism).

The ‘New Arctic’ is positioned in the Anthropocene, which also comes with digital data and the internet (for International Polar Years IPYs see^19^ and^1^; examples shown in^20–22^. For this part of the world obtaining publicly available primary data of ground and aerial surveys with supporting information—in a modern format—is very difficult despite some large species numbers and bird density and abundance^10^, though see some exceptions such as^13^. While environmental impact assessments are increasingly needed in the stressed area (e.g.^20^) and are to be based on best-available information^23^, internet resources such as GBIF.org or eBIRD are showing little data finds for this study area, nor are relevant bird banding data publically shared and accessible for this region, yet. All awhile, the majority of birds on those flyways are clearly declining^24^ (see^25^ for songbirds,^26^ for a highly endangered shorebird^27^, for Siberian crane^17^; for status of other geese; see^28^ for historic perspectives on geese declines in Siberia, Asian wintering grounds etc.). Wilderness habitats along the flyway are also in an alarming down-trend, while urbanization is on the rise (see^29^ for interior Asia flyway habitats). Excessive use of insecticides and pesticides, in addition to rampant poaching, has been described for many years^28,30–32^. Still, some A*nser* species are on the rebound and increase.

To set a baseline research conservation example for the ‘New Arctic’, here we focus on two key hunted goose species important for the local and indigenous people and their habitats of concern: Tundra Bean Goose *Anser fabalis serrirostris (*hereafter TuBEGO*; Taxonomic Serial Number TSN* from itis.gov 175024)*,* and Greater White-fronted Goose *A. albifrons TSN* 175020 (hereafter GWFG). These birds do nest in the high Arctic, stop-over in agriculture landscapes in the boreal zone and winter in natural and agriculture habitats in the East Asia^17,33,34^. According to^17^ TuBEGO is part of the ‘A4 Eastern Tundra Bean Goose *Anser fabalis serrirostris*’ population, whereas GWFG is part of the ‘C3 Tule Greater White-fronted Goose *Anser albifrons elgasi*’ population. We consider populations attributed to the West Pacific branch of the East-Asian-Australasian Flyway (see definition of this branch in^33,34^ because these populations are known to have increased for both species through global change and due to a switch to agricultural habitats on wintering grounds in Korea and Japan^35,36^ for rough estimates of wintering grounds)). Many of those areas are coastal with agriculture, and the specific human populations are on the increase.

These species are of relevance because their arctic breeding grounds are still virtually free of a dense road network^37^ or settlements; however recent proposals for the developing of mining and infrastructure (including supporting roads) may be critical for impacting summer habitats of both geese species (see http://ecoline-eac.com/proekty/peschanka/deposit.html). While the arctic grazing systems are often considered pristine, they are not due to the overgrazing by abundant domestic reindeer^15,38,39^. And man-made climate change and associated permafrost thawing, and even fires add other man-made features now. The Anthropocene -its characteristics and problems—is clearly found in the Arctic and Arctic plain^40^ and along the species flyway^25^; it’s the ‘New Arctic’^2^ which also happens to be digital^1,20^, can use Machine Learning methods^18,20,27^ and has such processes, interactions and opportunities^23^.

Recent summer distribution data for these species—explicit in time and space^41^—are lacking, are not compiled and are not available in a good useable or digital format with metadata to understand them, thus far (compare with^20^). A subsequent open access model prediction for these species and their specific metrics does not yet exist but can be powerful for progress (e.g.^42,43^; but see^27^ for Siberian Crane^44^, for Lesser White-fronted Goose; and^45^ for concepts and workflows).

Using these Arctic geese allows for progress on this topic, which is important while development and massive changes push northwards and into the interior wilderness areas of this world and into the New Arctic^38^ (for status see^1,37^). A solid study for those two species and Open Access baseline can help here to set the stage, document and address conservation problems in a best-available scientific manner for betterment along the flyway, in the stop-over and wintering areas, as well as for protected area questions (e.g.^46^). A distribution model quantifies bird-habitat relationships for the area for those geese for the first time, and might be helpful in forecasts of changes related to climate and other drivers of populations^18^.

## Methods

### Workflow

**Figure 2:**
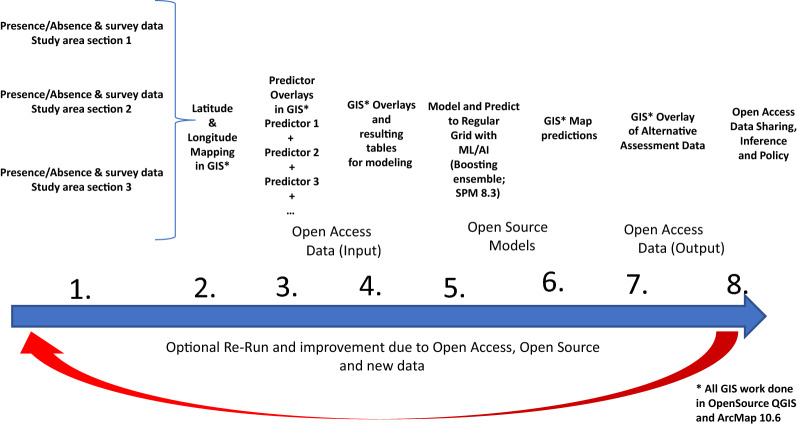
Workflow of this study.

Following best-practice^41^ (see^45^ as well as^20,27^ for applications) we developed a workflow. It shows how the data can be compiled, cleaned, employed in GIS and model predicted, subsequently assessed for performance, and be used and interpreted for inference; general model details and concepts are shown in^18^ and Fig. 2.

### Bird data

#### Model training data

The Russian Arctic is vast, and it presents the biggest landmass for this circumpolar ecosystem—arctic, boreal, terrestrial and marine alike^27,37,46^. Despite many decades of publications and efforts, our study area located in the Eastern Arctic with an extent of c. 3000 km East–West and c. 2500 km North–South is still poorly and not systematically surveyed for its birds with no centralized database readily available for geese species or their habitats and for specifics of breeding and post-breeding times. A coordinated research design or species atlas with models does not yet exist for this region (compare with^47^ for Yukon^48^, and^49^ for Alaska, or for Sweden see: https://www.ebba2.info/contribute-with-your-data/national-coordinators/sweden/; https://pecbms.info/country/sweden/).

In the absence of such data, for the first time we were attempt to compile representative area species sampling data sets for the two study species in their summer grounds. We focused on post-breeding, namely brood-rearing, and moulting time—mostly July–August—which has never really been spatially described, yet in a coherent fashion. Field data were collected during surveys performed by authors along the rivers and on lakes in the tundra during the period of 1997–2020 using motor-boat and aerial surveys. Visual surveys from moving motor-boat, foot ground surveys around lakes and aerial surveys were combined (Table 1). Only data on the presence and number of flightless geese, (moulting adults and brood-rearing groups) were used for this study. The flightless period is a critical time in the annual cycle of geese^50,51^. Their habitat requirements include food availability and safety from predators and people. That habitat requirement exists because flightless geese traditionally co-evolved with, and were hunted by, people for centuries. And during their wing-moult they are extremely sensitive to all kinds of human-induced disturbance. Being flightless, the geese stay stationary during that moulting period, and thus, their spatial distribution does not really change at least for one month except for minor local small-distance movements.

**Table 1:** Description and details of data used for the relative index of occurrence (RIO) in this study for two goose species. Subregions are shown in Fig. 1.

Species and sample size details	Location and subregion in Study Area	Survey focus	Source	Time period	Observer	Comment
**(a) Training data**						
*Anser albifrons (n* = *219; Broods present* = *142, Broods absent* = *77; Non-breeders present* = *95 Non-breeders absent* = *124)*	Yakutia	Brood & Non-breeders	Boat	2017, 2018	IB	
	Chaun	Brood & Non-breeders	Boat	2002 till 2019	DS, AK	
	Chukotka	Brood & Non-breeders	Aerial	2002,	AK	
	Chukotka: Koryak Highland	Brood & Non-breeders	Aerial	1997	AK	
*Anser fabalis (n* = *593; Broods present* = *213, Broods absent* = *380; Non-breeders present* = *303, Non-breeders absent* = *290)*	Yakutia	Brood & Non-breeders	Boat	2017, 2018	IB	
	Chaun	Brood & Non-breeders	Boat	2002 till 2019	DS, AK	
	Chukotka	Brood & Non-breeders	Aerial	2002,	AK	
	Chukotka: Koryak Highland	Brood & Non-breeders	Aerial	1997	AK	

Species	Source	Match with predictive model	Comment			
**(b) Testing data**						
*Anser albifrons*	GBIF presence (n = 63)	Good	These data are ‘just’ occurrences but indicate presence absence in the landscape			
	Compiled literature presence absence (n = 14)	Good	These data are also ‘just’ presence/absence but carry some attributes on broods and non-breeders (not shown here). The source is from the literature and first-time presented as a GIS layer			
*Anser fabalis*	GBIF presence (n = 17)	Good	These data are ‘just’ occurrences but indicate presence absence in the landscape			
	Compiled literature presence absence (n = 18)	Good	These data are also ‘just’ presence/absence but carry some attributes on broods and non-breeders (not shown here). The source is from the literature and first-time presented as a GIS layer			

The sampling data obtained during this geese census are a relative index of occurrence (RIO) and contain decimal latitude, decimal longitude, observation time (24 h) and date (day, month and year). We also included species presence and abundance, and we categorized birds as either: (1) moulting non-breeders or (2) breeding pairs with goslings. These data, two data sets for each of the two species, were put into an OpenSource datasheet using CSV format and were GIS-mapped (Fig. 1; Table 1). The surveyed areas and their rivers and lake areas are listed in Supplement 3.

#### Model assessment data

In addition to internal model metrics, it is important to assess models with alternative information, to compare models with reality^18,52,53^. Therefore, we used alternative cleaned data from GBIF.org (DOIs https://doi.org/10.15468/dl.up4kmu; https://doi.org/10.15468/dl.xwnkqe) and the literature to test our models (see Supplement 1). We also found other data sets like MOVEBANK, Bird Banding Center data and many research project data mentioned in publications but those were not available in an Open Access format and thus could not be used (*sensu*^19,41^).

### GIS data

Despite many decades of geological and geophysical survey work, modern GIS data layers for the study area are not really available, e.g. as needed as predictors in a raster format with known errors, a valid geographic projection and ISO-compliant metadata to understand them for a scientific purpose. We therefore followed data from Sriram and Huettmann (unpublished; https://essd.copernicus.org/preprints/essd-2016-65/) and added those open access layers as habitat predictors.

We selected GIS layers that are biologically meaningful or that are habitat use proxies and available for the prediction of the ecological niche for the two species during summer. Our models focus only on the Arctic tundra, and we used the CAVM map (https://www.caff.is/flora-cfg/circumpolar-arctic-vegetation-map) to exclude other habitat types where geese are not occurring, e.g. forests.

The following seven predictors were used for the study area: Global Landcover, Mean Temperature in July, Mean Precipitation in July, Annual NDVI, Human Footprint, Elevation (ETOPO1) and Human Density. A list of those GIS maps and their details can be seen in Supplement 2 and GIS files are available for free download and further use from sources mentioned.

### Data processing

We followed the workflow outlined in the beginning of this section (Fig. 2). We used ASCII CSV data and imported them into ArcGIS desktop 10.6 and OpenSource QGIS 3.16, and then overlaid them with GIS layers for the study area with external layout edits. The study area has a date line (180 degrees longitude located app. between Russia and Alaska). In addition, we used the Mercator geographic projection with a Pacific meridian using decimal latitude and longitude (WGS84). We then exported data from the GIS as a table for subsequent model-predictions presented in the next section. These steps are generally used in^20^ and in a more detailed way applied in^45^ and^20,27^^.^ as a proof of concept for the area.

### Predictive modeling

Here we are following a widely-used concept of inference from predictions^52,53^ (see^18,42^ for applications), employed for n-dimensional ecological niche models^20,42^. This was achieved by using Minitab-Salford Predictive Modeler (SPM 8.3; https://www.minitab.com/en-us/products/spm/). We employed TreeNet (Stochastic Gradient Boosting^18^; see^27^ for an application and example of the algorithm; see^42,43^ for general performance assessments of the algorithm as being among the most suited and powerful). To find the best solution we started with exploratory models and their metrics, e.g. confusion matrix and ROC, to be improved sequentially. We used default model settings (known to perform best^18^) with ‘balanced sampling weights’, tenfold Cross-Validation, a node depth of 10, and 2 as the minimum sample size for terminal tree branches; we used 400 trees to assure an optimal solution was found. Model diagnostics are presented in the appendices; see^18^ for general applications. As we employ non-parametric machine learning techniques we are less concerned with autocorrelation. Also, this is the first model of its kind and we did not emphasize specific questions of autocorrelation Stochastic Gradient boosting is robust to data with autocorrelation; for justification, conceptual details and lack of a problem see for instance^54^). After creating a grove file in SPM to capture the actual model in a software format, we scored an approx. 5 km point lattice and obtained pixel-based predictions. We used that conservative scale to overcome GIS data inaccuracies inherent in many of the currently available Arctic data, e.g. coastline location and digital elevation models (DEM). Those lattice points then were mapped for the study area and a GIS legend was fit to visualize the RIO.

### Model assessment

Our model was assessed in four ways for evidence: (i) Based on the 24 year presence and absence data we used an internal ROC of the exploratory models as readily provided by SPM and its confusion matrix^42,43^. (ii) For a deeper assessment we also overlaid the model surface with the training and absence data for each species and the two data sets (moulting non-breeders, broods) allowing for a visual assessment of the generalization achieved. (iii) We further used the alternative assessment data -GBIF.org and compiled (Russian) literature—which we also overlaid for a visual assessment of the predicted ecological niche for this species. Lastly, (iv) we then compared our findings with the literature and other sources for this species.

Overall, all of these four assessments allow us to get a generic confirmation of how well models perform, using all of the best publicly available data as lines of evidence.

### Ethical statement

All methods in this workflow and as presented were performed in accordance with the relevant guidelines, regulations and ethics committees by the authors and their institutions involved (see author list and affiliations for details). This research is entirely based on non-intrusive surveys, data compilation and digital data analysis; no specimen were collected. Field work was done in Russia remotely with binoculars and ‘naked eye’ in the field, following their national regulations accordingly. All presented map products and associated shapefiles were done with OpenSource QGIS 3.16 (https://www.qgis.org/en/site/forusers/download.html) and ArcGIS Desktop 10.6 under the University of Alaska Fairbanks (UAF) academic campus license by FH/EWHALE lab. The data processing and GIS work and edits were done in computer labs in Russia and Alaska/US and all applicable data are presented and available Open Access for a transparent and repeatable approach including ISO-compliant metadata.

## Results

We were able to compile for the first time find the best-available long-term data for the two species and their two metrics—brood locations and non-breeders—for the study area of Eastern Yakutia and Chukotka. Also, we were able to obtain the best publicly-available assessment data in a digital format explicit in space and time.

Further, our findings show the first achieved predictions and their assessments for post-breeding moult of Tundra Bean Goose and Greater White-fronted Goose for non-breeders and parents with brood (Table 2; Figs. 5 and 6).

**Table 2:** Predictor Importance Rank in model for two *Anser* species: brood-rearing parents with broods and moulting non-breeders (The top-2 predictors for each species strata are presented in bold.

Predictor name	Importance rank in model			
	Tundra Bean Goose: Non-breeders	Tundra Bean Goose: Brood	Greater White-fronted Goose: Non-breeders	Greater White-fronted Goose: Brood
Global Landcover	**85**	67	**93**	93
Mean Temperature in July	83	61	76	84
Mean Precipitation in July	60	**100**	**100**	90
Annual NDVI	82	65	89	**100**
Human Footprint	32	24	34	34
Elevation (ETOPO1)	**100**	**73**	92	**99**

Human population density shows little variation and relevance for the study area and is not shown).

### Species: Tundra Bean Goose

The moulting non-breeders are primarily distributed in coastal areas of Yakutia and Western Chukotka, thus inhabiting coastal plains. A low occurrence is predicted in the eastern study area, and the birds are more or less absent in the mountains of the interior Chukotka, wider inland and along the coast of northern Bering Sea (Fig. 5a). This shows a more nuanced and complex distributional picture than what was previously known; arguably, the distribution of this species is not as crisp as presented and assumed elsewhere.

For the parents with broods the above pattern shows even stronger, with the parents and broods primarily occurring in the western section of the study area. It is noteworthy that the parents with broods are absent along the coastline and are found more inland, primarily Yakutia Arctic and around the wider Chaun Bay region, while Chukotka Peninsula is widely free of this cohort (Fig. 6b).

It is noteworthy that the non-breeders are not really overlapping with the parents with broods; the latter concentrate in the western section of the study area and more inland.

### Species: Greater White-fronted Goose

The moulting non-breeders are widely dispersed in the study area but seem to avoid the mountain habitats, e.g. inner parts of the Chukotka Peninsula and parts of Yakutia.

For the actual parents with broods it shows an almost opposite pattern, where the species is found in the interior, specifically in Chukotka and in Yakutia.

The patterns are hardly overlapping and are somewhat complementary to each other. There are two distinct patches, leaving a coastal area free of this species.

### Model performance details and assessment

#### Model performance details

Our models achieved good to very good accuracy (details shown next section). Predictors most strongly center around an interaction between climatic metrics like summer precipitation, temperature, as well as elevation and landcover categories, added by NDVI (Table 2; detail shown in Supplement 5). While the human footprint showed a smaller role, those trends were upwards indicating that those geese are somewhat affiliated with the human footprint.

For Tundra Bean Goose broods in the multivariate context we identified NDVI as a powerful predictor with a positive relationship (Table 2; details shown in Supplement 5). Together with lower elevations below 150 m it indicates where brood-rearing habitats can be found in the study area. For non-breeders we found precipitation in July as a powerful predictor with a positive relationship (Table 2; details shown in Supplement 5). Together with specific arctic coastal landcover classes it indicates where moulting areas can be found in the study area.

For Greater White-fronted Goose broods we found precipitation in July as a powerful predictor, but with a negative relationship (Table 2; details shown in Supplement 5). Together with somewhat higher elevations around 300 m it indicates where brood-rearing geese occur in the study area. For non-breeders we identified elevation as a powerful1 4th predictor with a negative relationship (Table 2; details shown in Supplement 5). Together with specific landcover classes it indicates where moulting flocks can be found in the study area.

#### Model assessment details

**Figure 3:**
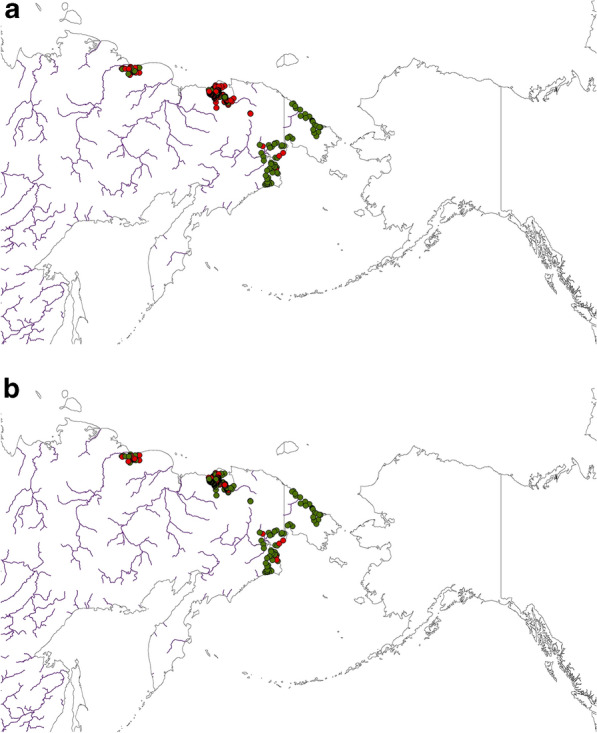
Best-available compiled raw data of Tundra Bean Goose *(Anser fabalis)* presence/absence for (**a**) Brood, and (**b**) Non-breeders in the study area. For both figures presence is shown in red and absence in green. Map created by FH with OpenSource QGIS and ArcGIS Desktop 10.6 academic license.

**Figure 4:**
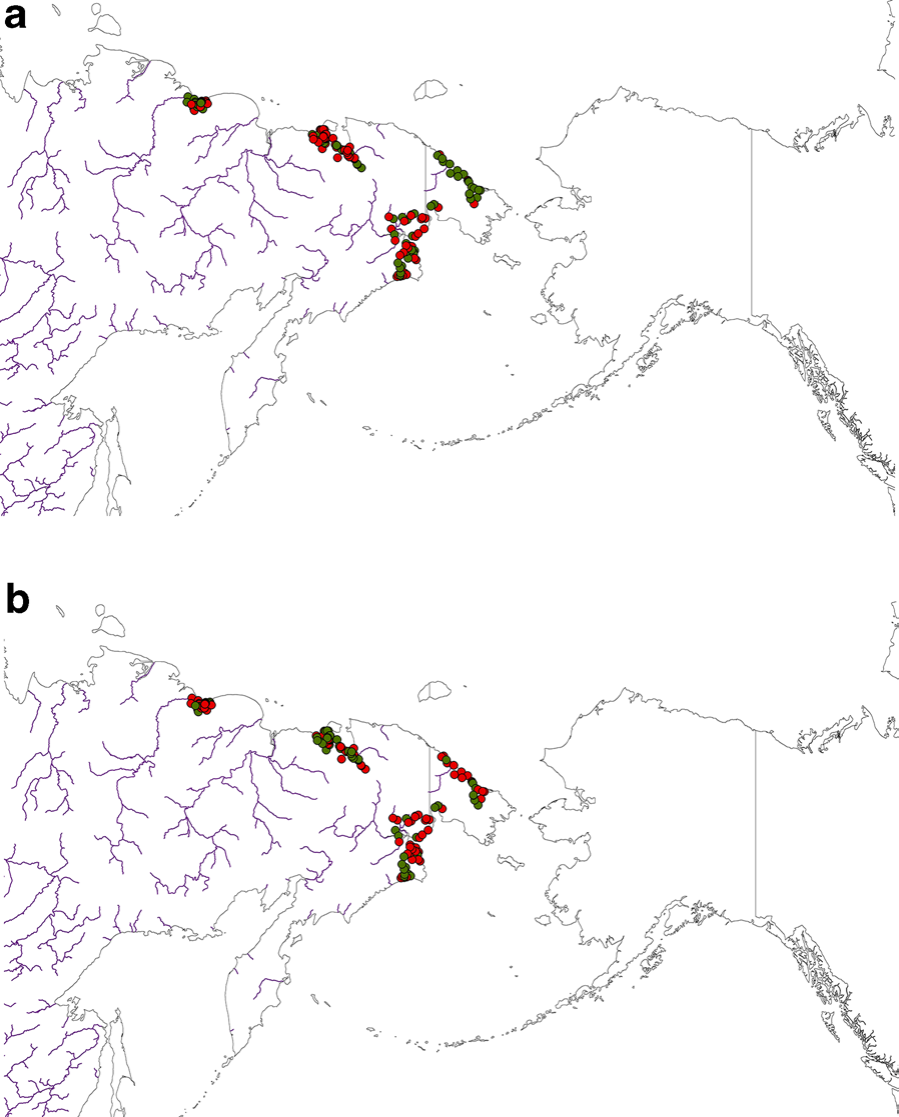
Best-available compiled raw data of Greater White-fronted Goose (*Anser albifrons)* presence/absence for (**a**) Brood, and (**b**) Non-breeders in the study area. For both figures presence is shown in red and absence in green. Map created by FH with OpenSource QGIS and ArcGIS Desktop 10.6 academic license.

**Figure 5:**
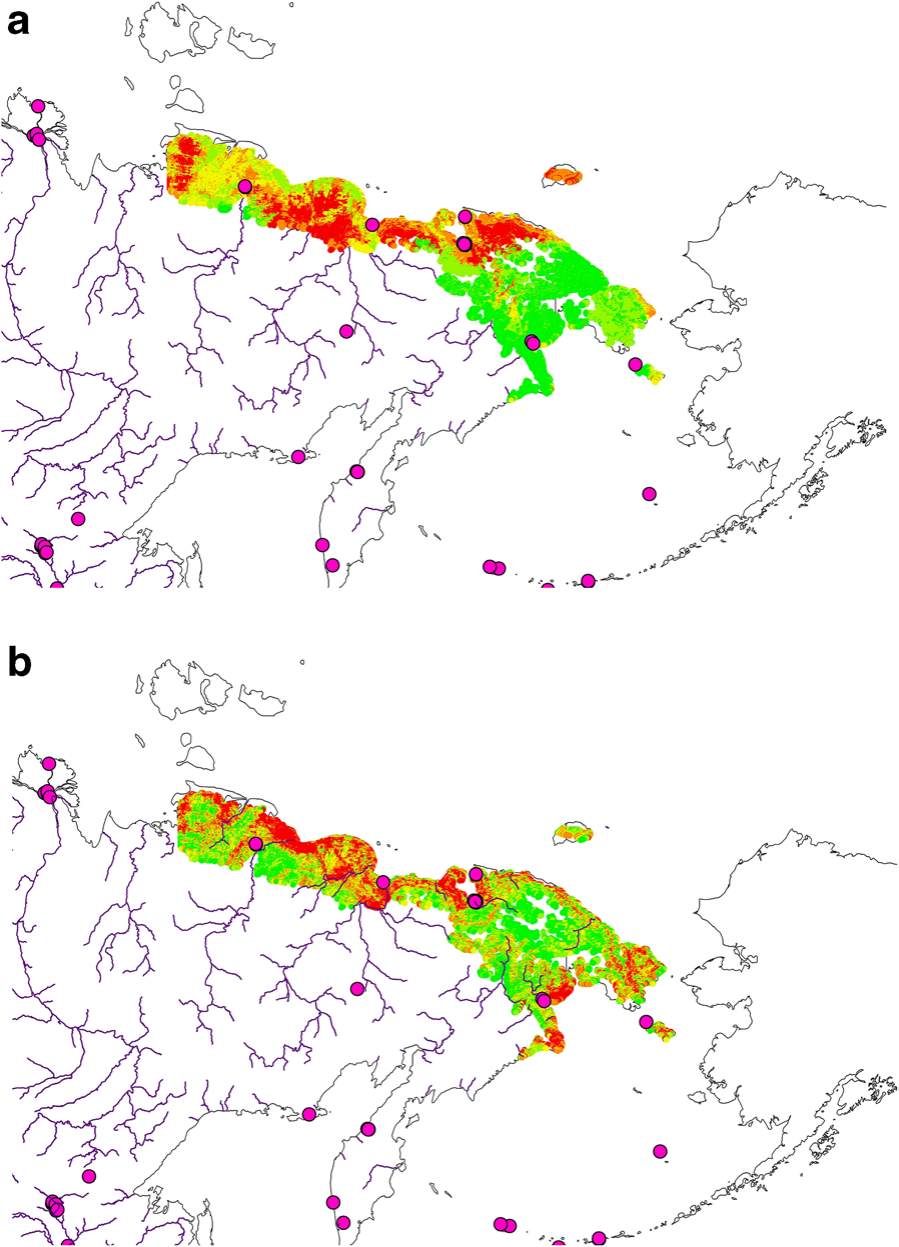
Predictions of Tundra Bean Goose (*Anser fabalis)* (**a**) brood-rearing parents with broods, and (**b**) post-breeding nonbreeders for the study area). The map predictions are presented as a ‘heatmap’ of the relative index of occurrence (RIO): red is a high RIO and green is a low RIO. Best-available GBIF presence location for this species are superimposed for assessment; they are shown as pink points. Map created by FH with OpenSource QGIS and ArcGIS Desktop 10.6 academic license.

**Figure 6:**
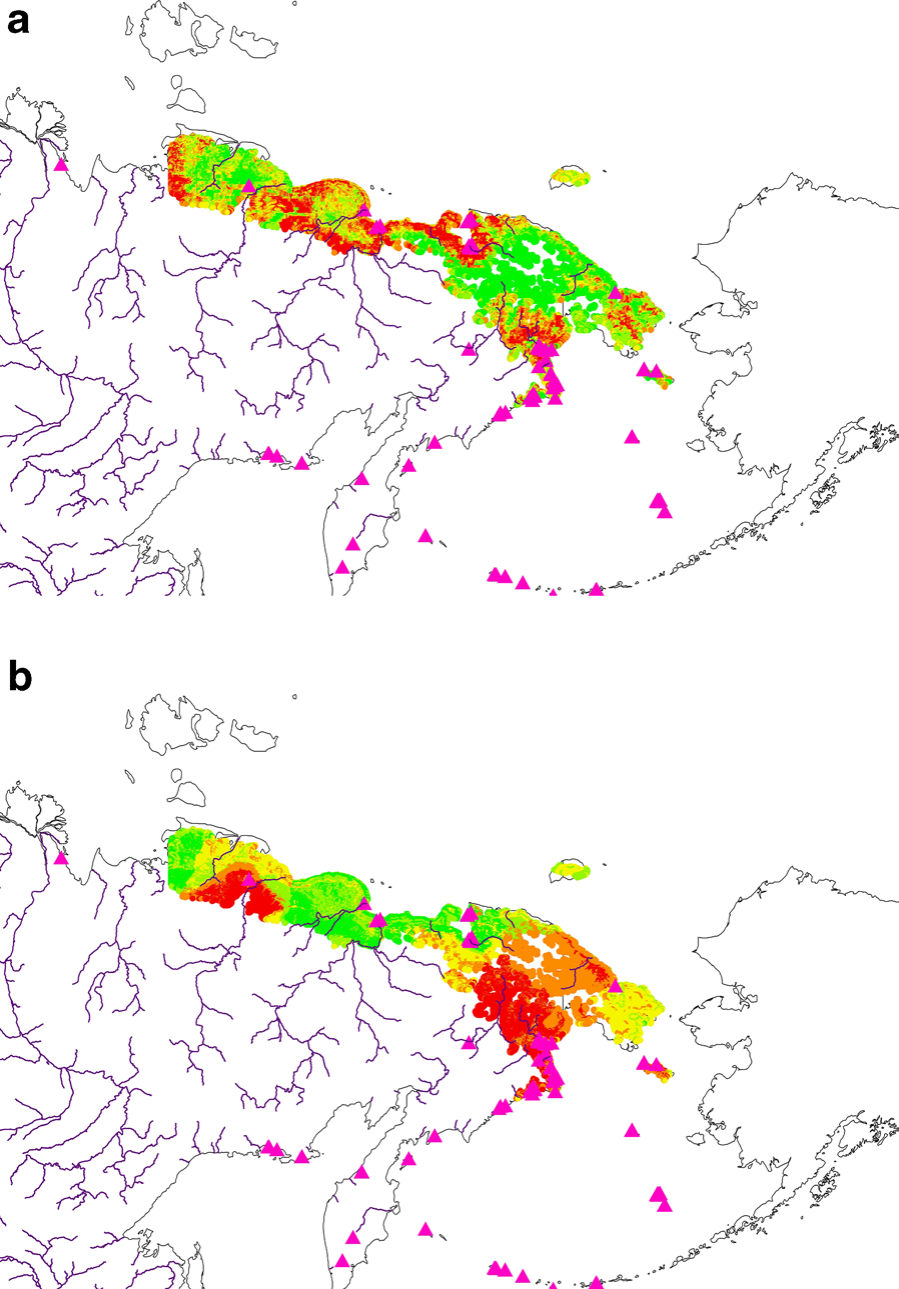
Predictions of Greater White-fronted Goose (*Anser albifrons*) (**a**) parents with broods, and (**b**) moulting nonbreeders for the study area). The map predictions are presented as a ‘heatmap’ of the relative index of occurrence (RIO): red is a high RIO and green is a low RIO. Best-available GBIF presence location for this species are overlaid for assessment; they are shown as pink points. Map created by FH with OpenSource QGIS and ArcGIS Desktop 10.6 academic license.

For robust inference and evidence, we actually used four pathways to assess the performance of our data-based model predictions for Tundra Bean Goose and Greater White-fronted Goose and their post-breeding non-breeders and parents with brood. The first is the internal aspatial ROC metric that comes with the exploratory model data itself. It shows a ROC of 82% (Tundra Bean Goose non-breeders*),* 85% (Tundra Bean Goose broods*)*, 91% (Greater White-fronted Goose non breeders) and 94% (Greater White-fronted Goose broods) for both species and their metrics. The ROC is based on the confusion matrix from the binary presence and pseudo-absence of the two survey data used for each of the two species (see Figs. 3, 4, 5 and 6). Those assessments indicate already a rather good model on the training data.

The second—more thorough—assessment is based on a visual match of the predictions with their training data on a map, allowing us to provide evidence of a good general match of the pattern predicted (see Figs. 5 and 6) for the two species and their metrics.

**Figure 7:**
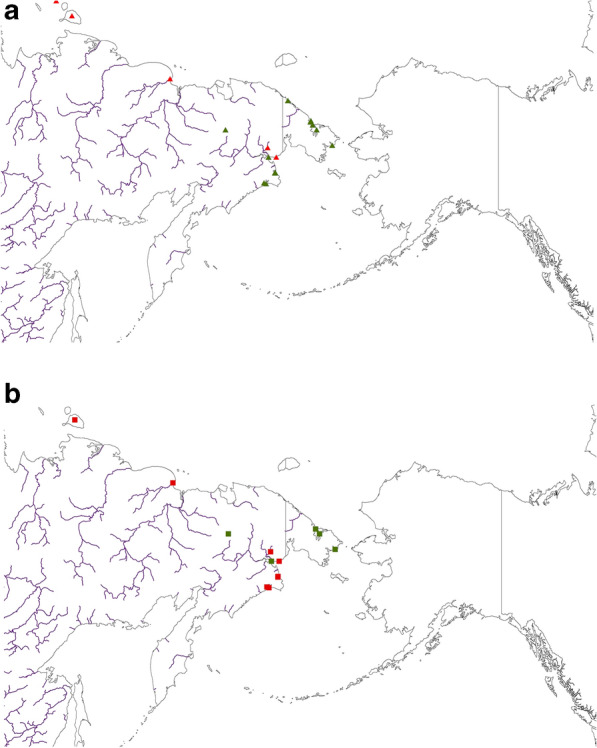
Best available occurrence data from the literature for (**a**) Tundra Bean Goose (*Anser fabalis;* shown as triangles) and (**b**) Greater White-fronted Goose (*Anser albifrons;* presented as squares. For both figures presence is shown in red and absence in green). Map created by FH with OpenSource QGIS and ArcGIS Desktop 10.6 academic license.

The third and fourth assessments, more independent but less specific for parents with broods and non-breeders, are based on the GBIF.org data and the compiled literature references for the species and its ecological niche overall in summer, less though for the brood and the non-breeders (see Table 1b; Fig. 7). But at least on a generic level it shows a very high match for the models (compare with Figs. 5, 6).

Taken the evidences together, overall, we therefore think that the methodology shown (Fig. 2 for workflow) and results presented are a good start for inference and offer us presentable validity, allowing to move next into thorough abundances and population trend models. Arguably, better data, e.g. more explicit, more extensive, and ideally corrected for detectability coming from a proper research design (see^55^ for an example) will allow for fine-tuning our findings further^1,2,39,40^.

## Discussion

For the study area of the Russian Eastern Arctic, this study is the first that compiled ISO-documented digital long-term data explicit in space and time for Tundra Bean Goose and Greater White-fronted Goose (Compare with^56^). While the true ranges remain unknown, here we provided important steps for two keystone species for the flyway. We tried to achieve the first digital model workflow and approaches for the species in Russia. We further tried to advance knowledge for this species by focusing on the post-breeding time and moult locations for (1) parents with broods, indicating also nesting habitats because non-flying goslings cannot move far from their nests, as well as (2) non-breeders away from the nest. Those data fill a gap in existing databases (e.g.^57^) and they are more specific than the generic ecological niche in summer and hand-drawn maps (e.g.^58,59^) for each species. In the wide absence of public information on these specific questions the data are part of the global arctic research legacy and the findings should be of good use and relevance for the study area and flyway as quantified baselines for bird monitoring, range estimates and subsequent population estimations and conservation management. Arguably, those deserve to be improved further and frequently with more data.

The habitat GIS layers are also the first of their kind compiled for this species for the public, the study area, provided in a modern digital grid format and made available free of charge in a documented form. Those data can be assessed and fine-tuned for more work as well (see Sriram and Huettmann unpublished for over 100 GIS layers to be used; see an application for the study area by^27^).

While this study has limitations, here we use an open access approach and we open all steps up for detailed review and scrutiny for model improvements; sensu ^53^.

Our models are the first generation of such workflows and deserve careful use. However, they are assessed with 4 lines of evidence and allow for a subsequent inference. They show us a new, nuanced and complex species distribution pattern. They have little overlap of parents with brood vs nonbreeders indicating movements and specific staging sites; it is a new piece of information and needs more study. This biological mismatch is most pronounced for Greater White-fronted Geese. It shows that non-breeders and probably early failed-breeders, stay apart from their breeding grounds commencing moult migration to the areas/habitats differing from the ones used by parents with broods. Generally, we found from our models that for both species’ parents with brood retreat from the coast and then move more inland. Except for non-or-failed- breeding Greater White-fronted Goose we found that Eastern Chukotka is of less relevance for both species during the post-breeding times. The Greater White-fronted Goose is distributed on both sides of the Bering Sea, being a truly circumpolar species, while Tundra Bean Goose is an Eurasian species, not existing in North America (replaced by the Canada goose).

However, despite the four lines of evidence matching these patterns our findings are previously unknown as they are only partly in agreement with the coarser^58,59^ maps and with^17^. In addition to showing more differentiated and realistic distribution patterns they also include highly preferable areas/habitats of populations migrating along the West Pacific Flyway.

In a multivariate context we found that climatic variables play a larger role for the presence of the two *Anser* species in post-nesting flightless times. We also found a positive relationship with NDVI (see also^60^ for green wave and NDVI link) and with the Human Footprint. However, Human Footprint is currently a weak predictor in our model probably because our study area is among the least populated in the world (some physical industrial footprint does exist though). Interestingly, in another Arctic nesting *Anser* species, the Lesser White-fronted Goose *Anser erythopus*, the habitat suitability in the same study area decreases with human disturbance, reflecting the negative impacts of human presence there. Lesser White-fronted Goose (the species is Endangered with declining population^17^) are found to select mostly human-free sites among huge area of suitable summer habitats^44^, while abundant and increasing Tundra Bean Goose and Greater White-fronted Goose are found to utilize a wider set of habitats including areas close to human settlements. Co-occurrence with humans may be an occasional result of selecting areas close to large and medium-sized rivers.

Overall, our predictions and assessments could have been stronger if existing data we located to exist were actually made better available by the international community^19,23^ see Table 3 for data that exist for the study area and study species).

**Table 3:** Data known to be available for the study area and of great use for this work but not publically made available or useable (Note: Best professional practices, the International Polar Year, Migrator Bird Treaties, and most national funding schemes make Open Access data sharing mandatory; see ^19^ for a reality assessment and as found here).

Data set name	Content	Source	Comment
Movebank	Geolocations	Various funders	Most data generally blocked behind login
Goose tagging	Locations	China	Most data generally not made publically available regardless of publication
Bird Banding	Banding location, resighting and recovery	National Bird Banding Center	EURING, nor the EU, is explitely not sharing geo-referenced data in GBIF
(International) expedition sighting records	Documented locations of presence and absence	Many researchers, institutions and NGOs worldwide	Those various data were collected and exist for over 50 years in the study area
*Citizen Science data	Documented locations of presence and absence	Many tourists, naturalists, governmental employees and researchers worldwide	iNaturalist, eBIRD etc. are growing rapidly

*Data are readily available but show little coverage and information for the study area, yet.

We would like to emphasize that our studied populations of both species are of the West Pacific Flyway, what it means is that their wintering areas are in Korea and Japan. Trends of Greater White-fronted Goose populations are contrasting between the West Pacific and East-Asian Continental Flyways, with the birds of the latter all wintering in China. However, from our work we feel that such strict delineations might be somewhat inaccurate, as the more graduated prediction maps show (see for instance^61–63^ for patterns). The inclusion of small-sized Lesser White-fronted Goose sharing summer and—in part—winter habitats with our study species poses another question of competition for the food resources to be studied in more detail^44^. More thought is to be given about their range, distribution and flyway memberships and ‘straddling’ while habitats and climates are changing so rapidly overthrowing evolved and assumed patterns.

**Figure 8:**
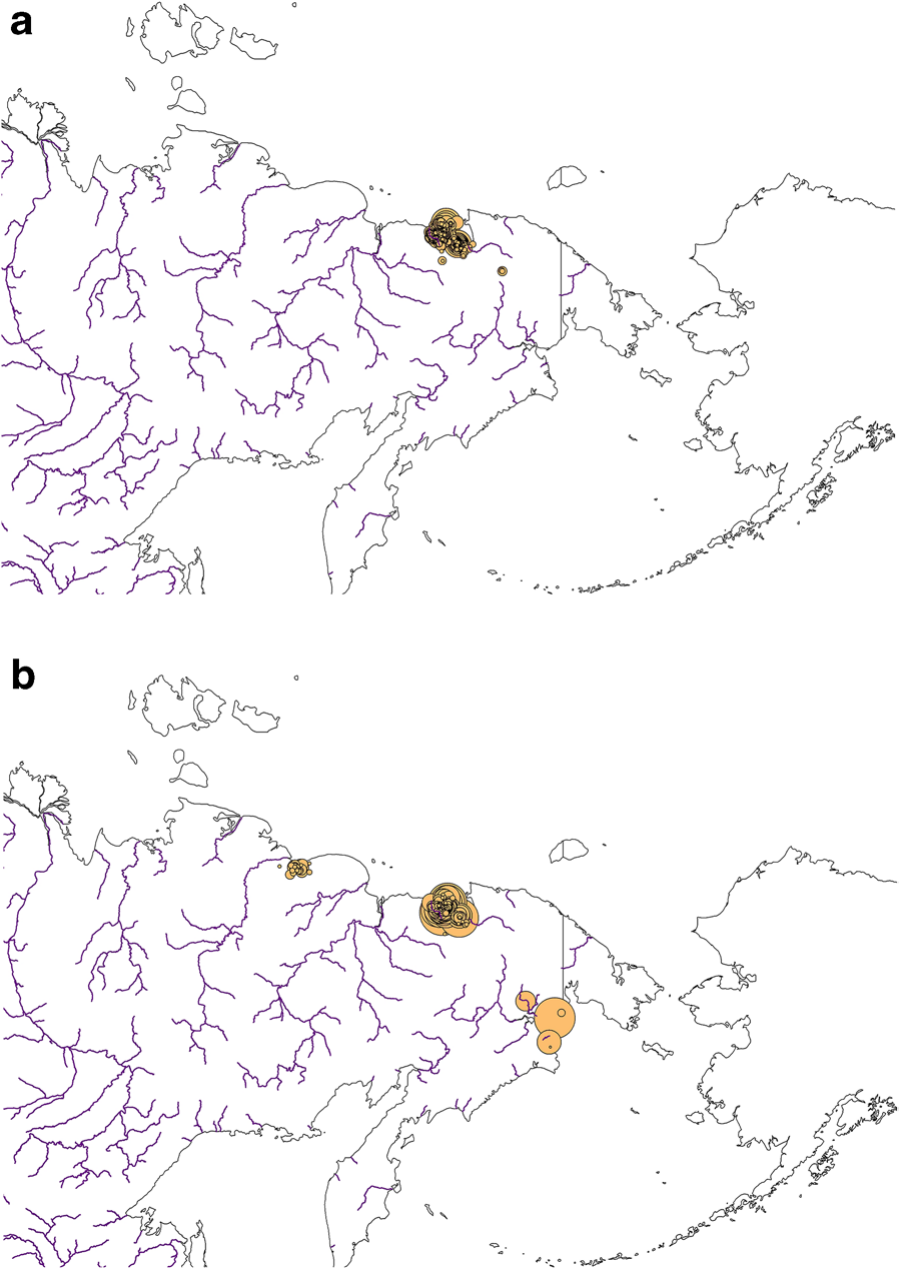
Field survey numbers for Tundra Bean Goose (*Anser fabalis*) (**a**) broods, and (**b**) moulting non-breeders for the study area). Yellow circles are scaled ranging from 0 to 100 individuals. Map created by FH with OpenSource QGIS and ArcGIS Desktop 10.6 academic license.

**Figure 9:**
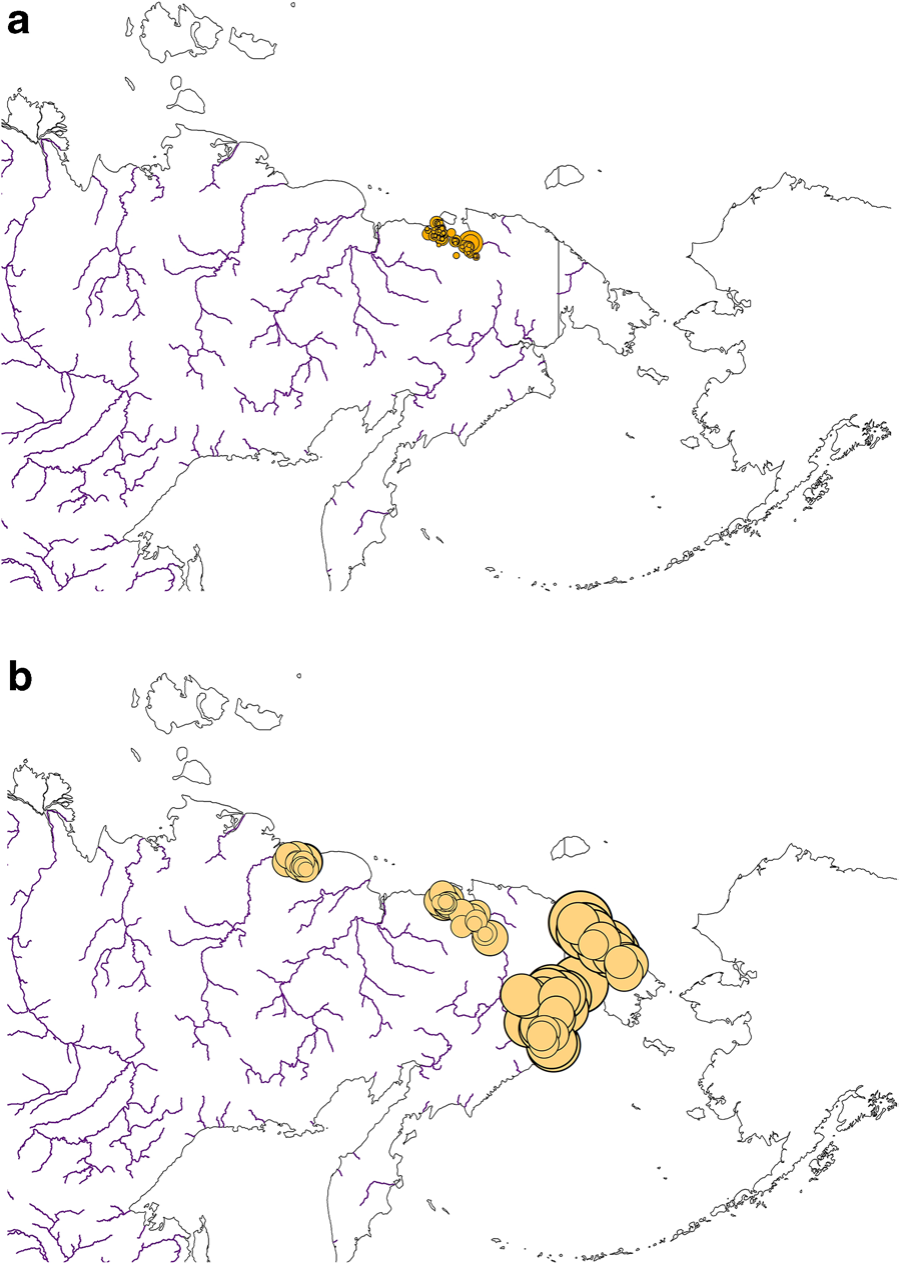
Field survey numbers for Greater White-fronted Goose (*Anser albifrons*) (**a**) broods, and (**b**) moulting non-breeders for the study area. Yellow circles are scaled ranging from 0 to 100 individuals (in **b**) a log scale was used ranging from 0 to 10,000). Map created by FH with OpenSource QGIS and ArcGIS Desktop 10.6 academic license.

In forthcoming work species abundances could be addressed to match for instance the overall flyway and winter estimates (for model concepts see^63,65^). But Figs. 8 and 9 make it clear from our additional survey data we compiled that numbers seem to be large when extrapolated to the ecological niche that we presented here.

As presented by^27^ we find that approaches of data mining, predictive modeling done with an open access and open source concept are new, very promising, insightful and should be applied here more and with policy implementations. However, changes that are currently happening in the Arctic and its flyways are dramatic, shaping global processes and events, and it is unclear whether concerted policy actions even can mitigate them any time soon.

These findings matter because they help filling study gaps in time and space, as well showing the state of the art for these species, their habitats and scientific data. It is noteworthy that the species studied are also vectors for diseases, which in times of pandemics are of importance (e.g.^64,65^).

Lastly, and as shown in^20,25^ it should be feasible to create circumpolar and/or flyway predictions for the species of interest in order to tackle modern questions of Arctic and migratory species management. These predictions can be high resolution explicit in time, in space and in the biology, e.g. for subspecies, timings and physiology, as it was started here (see^66^ for high resolution model options). While no meaningful large-scale tracking of high arctic species and ecological niche estimates exist yet, those data from Movebank, Bird banding and other efforts—if made publicly available—would contribute much to all efforts reported here, ideally, for future predictions during a still unabated man-made climate change with associated sustainable policy implications.

## Supplementary Information

Supplementary Information 1.

Supplementary Information 2.

Supplementary Information 3.

Supplementary Information 4.

Supplementary Information 5.

Supplementary Information 6.

Supplementary Information 7.

Supplementary Information 8.

Supplementary Information 9.

Supplementary Information 10.

Supplementary Information 11.
