# Supplementary material for: Modeling Eastern Russian High Arctic Geese (Anser fabalis, A. albifrons) during moult and brood rearing in the ‘New Digital Arctic’
Source: Sci Rep. 2021 Nov 11;11:22051. doi: 10.1038/s41598-021-01595-7 (PMC8586028; doi:10.1038/s41598-021-01595-7)
Supplement: Supplementary file 1 — Supplementary Information 1. [file 41598_2021_1595_MOESM1_ESM.html]

Complied 24 years (1997-2020) survey and model assessment data sets for the Eastern Russian Arctic for brood rearing, moulting, presence/absence and abundance indeces of Tundra Bean Goose (Anser fabalis) and Greater White-fronted Goose (Anser albifrons),


# Complied 24 years (1997-2020) survey and model assessment data sets for the Eastern Russian Arctic for brood rearing, moulting, presence/absence and abundance indeces of Tundra Bean Goose (Anser fabalis) and Greater White-fronted Goose (Anser albifrons),

Metadata also available as

### Metadata:

- Identification\_Information
- Data\_Quality\_Information
- Entity\_and\_Attribute\_Information
- Distribution\_Information
- Metadata\_Reference\_Information

Identification\_Information:

Citation:

Citation\_Information:

Originator:

Diana Solovyeva, Inga Bysykatova-Harmey, Sergey L. Vartanyan, N. A. Shilo, Alexander Kondratyev, Falk Huettmann

Publication\_Date: 20210614  
Title:

Complied 24 years (1997-2020) survey and model assessment data sets for the Eastern Russian Arctic for brood rearing, moulting, presence/absence and abundance indeces of Tundra Bean Goose (Anser fabalis) and Greater White-fronted Goose (Anser albifrons),

Edition: 1  
Geospatial\_Data\_Presentation\_Form: vector digital data  
Other\_Citation\_Details: See manuscript on the topic coming forward.  
Online\_Linkage: https://doi.org/10.5066/xxxxxxxx

Description:

Abstract:

This dataset is the best-available public presence information for brood rearing and moutling Tundra Bean Goose (Anser fabalis) and Greater White-fronted Goose (Anser albifrons) for the Russian Eastern Arctic (Yakutia and Chukotka), These data were collected by the Russian co-authors from 1997 til 2020 in the field on foot, by aircraft and motor boat. These data also include abundance estimate indeces for brood rearing and moulting birds.For A. albifrons n=219 there were 77 absences of brood rearing and 142 presences of brood rearing, and 124 absences of moulting and 95 presences of moulting.
For A. fabialis n=593 there were 380 absences of brood rearing and 213 presences of brood rearing, and 290 absences of moulting and 303 presences of moulting.
Additionally,less detailed,l data are compiled from the literature (n=14 for A. albifrons, n=18 for A. fabalis; 2002-2018) and from GBIF.org (n=63 for A. albifrons, n=17 for A. fabalis).. All of these datasets are geo-referenced with latitude and longitude, geographic projection of WGS84. Some of the data came initially from different projections.
The study area of the data coverage features the international dateline and several national projections were initially used for the GIS data.

Purpose:

Presence and some abundance modeling for the Russian Eastern Arctic.

Supplemental\_Information:

There is a manuscript by the authors that explains the data use, and more details.

Time\_Period\_of\_Content:

Time\_Period\_Information:

Range\_of\_Dates/Times:

Beginning\_Date: 19970601  
Ending\_Date: 20200831

Currentness\_Reference: ground condition

Status:

Progress: Complete  
Maintenance\_and\_Update\_Frequency: None planned

Spatial\_Domain:

Description\_of\_Geographic\_Extent: World  
Bounding\_Coordinates:

West\_Bounding\_Coordinate: -175.7813  
East\_Bounding\_Coordinate: 180.0000  
North\_Bounding\_Coordinate: 90.0000  
South\_Bounding\_Coordinate: 62.2679

Keywords:

Theme:

Theme\_Keyword\_Thesaurus: ISO 19115 Topic Category  
Theme\_Keyword: biota

Theme:

Theme\_Keyword\_Thesaurus: None  
Theme\_Keyword: Greater White-fronted Goose (Anser albifrons),  
Theme\_Keyword: Tundra Bean Goose (Anser fabalis)  
Theme\_Keyword: Moulting  
Theme\_Keyword: Brood rearing  
Theme\_Keyword: Russian Eastern Arctic  
Theme\_Keyword: Yakutia  
Theme\_Keyword: Chukotka  
Theme\_Keyword: Literature references  
Theme\_Keyword: GBIF.org

Place:

Place\_Keyword\_Thesaurus: None  
Place\_Keyword:

The associated manuscript has an appendix with english place names where the surveys were carried out.

Taxonomy:

Keywords/Taxon:

Taxonomic\_Keyword\_Thesaurus: None  
Taxonomic\_Keywords: Anser albifrons  
Taxonomic\_Keywords: Anser fabalis

Taxonomic\_Classification:

Taxon\_Rank\_Name: Kingdom  
Taxon\_Rank\_Value: Animalia  
Applicable\_Common\_Name: animals  
Taxonomic\_Classification:

Taxon\_Rank\_Name: Subkingdom  
Taxon\_Rank\_Value: Bilateria  
Taxonomic\_Classification:

Taxon\_Rank\_Name: Infrakingdom  
Taxon\_Rank\_Value: Deuterostomia  
Taxonomic\_Classification:

Taxon\_Rank\_Name: Phylum  
Taxon\_Rank\_Value: Chordata  
Applicable\_Common\_Name: chordates  
Taxonomic\_Classification:

Taxon\_Rank\_Name: Subphylum  
Taxon\_Rank\_Value: Vertebrata  
Applicable\_Common\_Name: vertebrates  
Taxonomic\_Classification:

Taxon\_Rank\_Name: Infraphylum  
Taxon\_Rank\_Value: Gnathostomata  
Taxonomic\_Classification:

Taxon\_Rank\_Name: Superclass  
Taxon\_Rank\_Value: Tetrapoda  
Taxonomic\_Classification:

Taxon\_Rank\_Name: Class  
Taxon\_Rank\_Value: Aves  
Applicable\_Common\_Name: Birds  
Taxonomic\_Classification:

Taxon\_Rank\_Name: Order  
Taxon\_Rank\_Value: Anseriformes  
Applicable\_Common\_Name: Ducks  
Applicable\_Common\_Name: Geese  
Applicable\_Common\_Name: Screamers  
Applicable\_Common\_Name: Swans  
Applicable\_Common\_Name: Waterfowl  
Taxonomic\_Classification:

Taxon\_Rank\_Name: Family  
Taxon\_Rank\_Value: Anatidae  
Applicable\_Common\_Name: Ducks  
Applicable\_Common\_Name: Geese  
Applicable\_Common\_Name: Swans  
Taxonomic\_Classification:

Taxon\_Rank\_Name: Subfamily  
Taxon\_Rank\_Value: Anserinae  
Taxonomic\_Classification:

Taxon\_Rank\_Name: Genus  
Taxon\_Rank\_Value: Anser  
Applicable\_Common\_Name: Bean Geese  
Taxonomic\_Classification:

Taxon\_Rank\_Name: Species  
Taxon\_Rank\_Value: Anser albifrons  
Applicable\_Common\_Name: Greater White-fronted Goose  
Applicable\_Common\_Name: TSN: 175020

Taxonomic\_Classification:

Taxon\_Rank\_Name: Species  
Taxon\_Rank\_Value: Anser fabalis  
Applicable\_Common\_Name: Bean Goose  
Applicable\_Common\_Name: TSN: 175024

Access\_Constraints: None. Please see 'Distribution Info' for details.  
Use\_Constraints:

None. Users are advised to read the dataset's metadata thoroughly to understand appropriate use and data limitations.

Point\_of\_Contact:

Contact\_Information:

Contact\_Person\_Primary:

Contact\_Person: Falk Huettmann  
Contact\_Organization: -EWHALE lab-

Contact\_Position: Professor  
Contact\_Address:

Address\_Type: mailing and physical  
Address: 2140 North Koyukuk Drive  
City: Fairbanks  
State\_or\_Province: Alaska  
Postal\_Code: 99775  
Country: US

Contact\_Voice\_Telephone: +1 907 474 7882  
Contact\_Electronic\_Mail\_Address: fhuettmann@alaska.edu

Data\_Set\_Credit: see authors and sources, e.g. GBIF and literature  
Native\_Data\_Set\_Environment:

MS Excel, transferred to ESRI shapefile from various projections into singular data layers.

Data\_Quality\_Information:

Attribute\_Accuracy:

Attribute\_Accuracy\_Report:

Data are compiled from various notebooks and field work 'as is'.

Logical\_Consistency\_Report:

Data are collected consistently with the same methods over the years.

Completeness\_Report: Data are complete  
Positional\_Accuracy:

Horizontal\_Positional\_Accuracy:

Horizontal\_Positional\_Accuracy\_Report:

Latitude and longitude were provided with a GPS, app 4-6 decimals

Vertical\_Positional\_Accuracy:

Vertical\_Positional\_Accuracy\_Report: NA

Lineage:

Process\_Step:

Process\_Description:

Data were compiled during the field, written into field notebooks and then put into MS Excel by the respective Russian authors.
FH compiled the data and put them into shapefiles with the help of the co-authors.

Process\_Date: 20210501

Entity\_and\_Attribute\_Information:

Overview\_Description:

Entity\_and\_Attribute\_Overview:

Data follow generally the x,y and z format, where latitude, longitude and attributes are provided. The 24 year data set also features abundance information for brood rearing geese as well as moulting ones. The GBIF.org data have a wider column format of Darwin Core..

Entity\_and\_Attribute\_Detail\_Citation:

Distribution\_Information:

Distributor:

Contact\_Information:

Contact\_Person\_Primary:

Contact\_Person: Falk Huettmann  
Contact\_Organization: -EWHALE lab-

Contact\_Position: Professor  
Contact\_Address:

Address\_Type: mailing and physical  
Address: 2104 North Koyukuk Drive  
City: Fairbanks  
State\_or\_Province: Alaska  
Postal\_Code: 99775  
Country: United States

Contact\_Voice\_Telephone: 1-888-275-8747  
Contact\_Electronic\_Mail\_Address: fhuettmann@alaska.edu

Distribution\_Liability:

Unless otherwise stated, all data, metadata and related materials are considered to satisfy the quality standards relative to the purpose for which the data were collected. Although these data and associated metadata have been reviewed for accuracy and completeness and approved for release by the U.S. Geological Survey (USGS), no warranty expressed or implied is made regarding the display or utility of the data on any other system or for general or scientific purposes, nor shall the act of distribution constitute any such warranty.

Custom\_Order\_Process: Contact authors, see journal manuscript

Metadata\_Reference\_Information:

Metadata\_Date: 20210614  
Metadata\_Contact:

Contact\_Information:

Contact\_Person\_Primary:

Contact\_Person: Falk Huettmann  
Contact\_Organization: -EWHALE lab-

Contact\_Position: Professor  
Contact\_Address:

Address\_Type: mailing and physical  
Address: Biology & Wildlife Dept. Inst of Arctic Biology  
City: Fairbanks  
State\_or\_Province: Alaska  
Postal\_Code: 99775  
Country: USA

Contact\_Voice\_Telephone: +1 907 474 7882  
Contact\_Electronic\_Mail\_Address: fhuettmann@alaska.edu

Metadata\_Standard\_Name:

FGDC Biological Data Profile of the Content Standard for Digital Geospatial Metadata

Metadata\_Standard\_Version: FGDC-STD-001.1-1999

---

Generated by mp version 2.9.50 on Thu Jun 17 23:12:34 2021
