# Supplementary material for: Modeling Eastern Russian High Arctic Geese (Anser fabalis, A. albifrons) during moult and brood rearing in the ‘New Digital Arctic’
Source: Sci Rep. 2021 Nov 11;11:22051. doi: 10.1038/s41598-021-01595-7 (PMC8586028; doi:10.1038/s41598-021-01595-7)
Supplement: Supplementary file 4 — Supplementary Information 4. [file 41598_2021_1595_MOESM4_ESM.docx]

Supplement 2. GIS Data with ISO-compliant metadata

List of six predictors and tundra mask

| **Predictor Name** | **Content** | **Source and detail** |
| --- | --- | --- |
| Global Landcover | Landcover map | See Sriram and Huettmann (unpublished online) |
| Mean Temperature in July | Long-term multi-year mean temperature for July in Degrees Celsius | See Sriram and Huettmann (unpublished online) |
| Mean Precipitation in July | Long-term multi-year mean precipitation for July in millimeters | See Sriram and Huettmann (unpublished online) |
| Annual NDVI | Normalized Difference Vegetation Index | See Sriram and Huettmann (unpublished online) |
| Human Footprint | Human footprint on the landscape | See Sriram and Huettmann (unpublished online) |
| Elevation (ETOPO1) | Elevation above sea level in meters | See Sriram and Huettmann (unpublished online) |
| Circumpolar Arctic Vegetation Map (CAVM) | Vegetation map for the circumpolar Arctic | CAVM |
