# Supplementary material for: Modeling Eastern Russian High Arctic Geese (Anser fabalis, A. albifrons) during moult and brood rearing in the ‘New Digital Arctic’
Source: Sci Rep. 2021 Nov 11;11:22051. doi: 10.1038/s41598-021-01595-7 (PMC8586028; doi:10.1038/s41598-021-01595-7)
Supplement: Supplementary file 5 — Supplementary Information 5. [file 41598_2021_1595_MOESM5_ESM.html]

Complied GIS habitat data layers for the Eastern Russian Arctic for geese- and wildlife-related habitat models


# Complied GIS habitat data layers for the Eastern Russian Arctic for geese- and wildlife-related habitat models

Metadata also available as

### Metadata:

- Identification\_Information
- Data\_Quality\_Information
- Entity\_and\_Attribute\_Information
- Distribution\_Information
- Metadata\_Reference\_Information

Identification\_Information:

Citation:

Citation\_Information:

Originator: Falk Huettmann  
Publication\_Date: 20210614  
Title:

Complied GIS habitat data layers for the Eastern Russian Arctic for geese- and wildlife-related habitat models

Edition: 1  
Geospatial\_Data\_Presentation\_Form: raster digital data  
Other\_Citation\_Details:

Sources is from Sriram and Huettmann (unpublished)
See manuscript on the topic coming forward.

Online\_Linkage: https://doi.org/10.5066/xxxxxxxx

Description:

Abstract:

This value-added dataset is the best-available public habitat GIS layer for the Russian Eastern Arctic (Yakutia and Chukotka), These data were compiled from Sriram and Huettmann (unpublished). The datasets consist of Mean July temperature and precipitation, NDVI, ETOPO1 DEM, human footprint, as well as human population. These datasets are geo-referenced rasters with latitude and longitude, geographic projection of WGS84. Some of the data came initially from different sources. The Circumpolar Arctic Vegetation Maps CAVM was used as a mask for the tundra. A coastline shapefiel in Mercator 180 degree central Pacific meridian was used for a study area template.

Purpose:

Presence and some abundance modeling for the Russian Eastern Arctic.

Supplemental\_Information:

There is a manuscript by the authors that explains the data use, and more details.

Time\_Period\_of\_Content:

Time\_Period\_Information:

Range\_of\_Dates/Times:

Beginning\_Date: 19970601  
Ending\_Date: 20200831

Currentness\_Reference: ground condition

Status:

Progress: Complete  
Maintenance\_and\_Update\_Frequency: None planned

Spatial\_Domain:

Description\_of\_Geographic\_Extent: World  
Bounding\_Coordinates:

West\_Bounding\_Coordinate: -175.7813  
East\_Bounding\_Coordinate: 180.0000  
North\_Bounding\_Coordinate: 84.7384  
South\_Bounding\_Coordinate: 62.2679

Keywords:

Theme:

Theme\_Keyword\_Thesaurus: ISO 19115 Topic Category  
Theme\_Keyword: biota

Theme:

Theme\_Keyword\_Thesaurus: None  
Theme\_Keyword: Temperature July  
Theme\_Keyword: Precipitation July  
Theme\_Keyword: NDVI  
Theme\_Keyword: ETOPO1 Digital Elevation Model  
Theme\_Keyword: World Coastline  
Theme\_Keyword: Rivers, major  
Theme\_Keyword: Global Landcover  
Theme\_Keyword: Human Footprint  
Theme\_Keyword: Circumpolar Arctic Vegetation Map

Place:

Place\_Keyword\_Thesaurus: None  
Place\_Keyword: Russian Eastern Arctic  
Place\_Keyword: Yakutia  
Place\_Keyword: Chukotka  
Place\_Keyword: Northern Kamchatka

Access\_Constraints: None. Please see 'Distribution Info' for details.  
Use\_Constraints:

None. Users are advised to read the dataset's metadata thoroughly to understand appropriate use and data limitations.

Point\_of\_Contact:

Contact\_Information:

Contact\_Person\_Primary:

Contact\_Person: Falk Huettmann  
Contact\_Organization: -EWHALE lab-

Contact\_Position: Professor  
Contact\_Address:

Address\_Type: mailing and physical  
Address: 2140 North Koyukuk Drive  
City: Fairbanks  
State\_or\_Province: Alaska  
Postal\_Code: 99775  
Country: US

Contact\_Voice\_Telephone: +1 907 474 7882  
Contact\_Electronic\_Mail\_Address: fhuettmann@alaska.edu

Data\_Set\_Credit:

see authors and sources, namely Sriram and Huettmann (unpublished)

Native\_Data\_Set\_Environment: See Sriram and Huettmann (unpublished)

Data\_Quality\_Information:

Attribute\_Accuracy:

Attribute\_Accuracy\_Report:

Data are compiled from Sriram and Huettmann unpublished and accurate from those sources

Logical\_Consistency\_Report: Data are compiled consistently with the same methods.  
Completeness\_Report: Data are complete  
Positional\_Accuracy:

Horizontal\_Positional\_Accuracy:

Horizontal\_Positional\_Accuracy\_Report:

Data came from geographic projection, latitude longitude, WGS84 in ArcGIS

Vertical\_Positional\_Accuracy:

Vertical\_Positional\_Accuracy\_Report: NA

Lineage:

Process\_Step:

Process\_Description:

Data came from Sriram and Huettmann (unpublished) and used in a WGS84 and mercator projection centered on the 180 degree Pacific.

Process\_Date: 20210501

Entity\_and\_Attribute\_Information:

Overview\_Description:

Entity\_and\_Attribute\_Overview:

Data follow generally the x,y and z format, where latitude, longitude and attributes are provided in a raster grid format (TIFF).

Entity\_and\_Attribute\_Detail\_Citation: See sources in Sriram and Huettmann unpublished.

Distribution\_Information:

Distributor:

Contact\_Information:

Contact\_Person\_Primary:

Contact\_Person: Falk Huettmann  
Contact\_Organization: -EWHALE lab-

Contact\_Position: Professor  
Contact\_Address:

Address\_Type: mailing and physical  
Address: 2104 North Koyukuk Drive  
City: Fairbanks  
State\_or\_Province: Alaska  
Postal\_Code: 99775  
Country: United States

Contact\_Voice\_Telephone: 1-888-275-8747  
Contact\_Electronic\_Mail\_Address: fhuettmann@alaska.edu

Distribution\_Liability:

Unless otherwise stated, all data, metadata and related materials are considered to satisfy the quality standards relative to the purpose for which the data were collected. Although these data and associated metadata have been reviewed for accuracy and completeness and approved for release by the U.S. Geological Survey (USGS), no warranty expressed or implied is made regarding the display or utility of the data on any other system or for general or scientific purposes, nor shall the act of distribution constitute any such warranty.

Custom\_Order\_Process: Contact author, see journal manuscript forthcoming

Metadata\_Reference\_Information:

Metadata\_Date: 20210614  
Metadata\_Contact:

Contact\_Information:

Contact\_Person\_Primary:

Contact\_Person: Falk Huettmann  
Contact\_Organization: -EWHALE lab-

Contact\_Position: Professor  
Contact\_Address:

Address\_Type: mailing and physical  
Address: Biology & Wildlife Dept. Inst of Arctic Biology  
City: Fairbanks  
State\_or\_Province: Alaska  
Postal\_Code: 99775  
Country: USA

Contact\_Voice\_Telephone: +1 907 474 7882  
Contact\_Electronic\_Mail\_Address: fhuettmann@alaska.edu

Metadata\_Standard\_Name:

FGDC Biological Data Profile of the Content Standard for Digital Geospatial Metadata

Metadata\_Standard\_Version: FGDC-STD-001.1-1999

---

Generated by mp version 2.9.50 on Thu Jun 17 23:15:19 2021
