# Supplementary material for: Modeling Eastern Russian High Arctic Geese (Anser fabalis, A. albifrons) during moult and brood rearing in the ‘New Digital Arctic’
Source: Sci Rep. 2021 Nov 11;11:22051. doi: 10.1038/s41598-021-01595-7 (PMC8586028; doi:10.1038/s41598-021-01595-7)
Supplement: Supplementary file 7 — Supplementary Information 7. [file 41598_2021_1595_MOESM7_ESM.docx]

Supplement 3. Survey Location names (following local names; survey coordinates presented in Supplement 1 GIS point data)

| **Survey location** | **Study area** | **Comment** |
| --- | --- | --- |
| Ayon Island | West Chukotka |  |
| Chaun Bay, sea | West Chukotka |  |
| Chaun River | West Chukotka |  |
| Chaun-delta | West Chukotka |  |
| Emukkuvnyan River | West Chukotka |  |
| Gnilushka River | West Chukotka |  |
| Gusinka River | West Chukotka |  |
| KolarvaamRiver | West Chukotka |  |
| Konevaam River | West Chukotka |  |
| Koz'mina River | West Chukotka |  |
| Kremyanka River | West Chukotka |  |
| Lake in Gnilushka River mouth | West Chukotka |  |
| Lake in Ngaglyeyngyveem River basin | West Chukotka |  |
| Lake in Rauchua River basin | West Chukotka |  |
| Lake in Teukool River basin | West Chukotka |  |
| Lake in Umkuveem River basin | West Chukotka |  |
| Lake on Kyttyk P | West Chukotka |  |
| Lakes in Utyykool River basin | West Chukotka |  |
| Lakes in Emukkuvnyan R basin | West Chukotka |  |
| Lakes in Kolarvaam River basin | West Chukotka |  |
| Lakes in Leluveem River basin | West Chukotka |  |
| Lakes in Olvegyrgyvaam River basin | West Chukotka |  |
| Lakes in Onmatgyr River basin | West Chukotka |  |
| Lakes in Pineyveem River basin | West Chukotka |  |
| Lakes in Rakvachan River basin | West Chukotka |  |
| Lakes in Teukoolkay River basin | West Chukotka |  |
| Lakes in Tikhaya River basin | West Chukotka |  |
| Lakes on Ayon Island | West Chukotka |  |
| Leluveem River | West Chukotka |  |
| Lishaynikovyi Creek | West Chukotka |  |
| Milgiveem River | West Chukotka |  |
| Mosey Island, lakes | West Chukotka |  |
| Ngagleyngyveem River | West Chukotka |  |
| Olvegyrgyvaam River | West Chukotka |  |
| Onmatgyr River | West Chukotka |  |
| Palyavaam channel | West Chukotka |  |
| Pineyveem River | West Chukotka |  |
| Pucheveem Channel | West Chukotka |  |
| Pucheveem River | West Chukotka |  |
| Rauchua River | West Chukotka |  |
| Rytkuchka River | West Chukotka |  |
| Sea coast Lesser Chaun Strait | West Chukotka |  |
| Sea near Rakvachan River | West Chukotka |  |
| Sea near Teukool River | West Chukotka |  |
| Sea near Ngagleynyn cape | West Chukotka |  |
| Senechkina Channel | West Chukotka |  |
| Teukool River | West Chukotka |  |
| Teukoolkay River | West Chukotka |  |
| Tikhaya River | West Chukotka |  |
| Umkuveem River | West Chukotka |  |
| Uttykool River | West Chukotka |  |
| Indigirka River | Yakutia |  |
| Yakutia  undefined river basin | Yakutia |  |
| Velikaya river basin | South Chukotka |  |
| Mountains in Koryak Highlands | South Chukotka |  |
| Kakanaut-Peulveem rivers, Koryak Highlands | South Chukotka |  |
| Gytkadalval river, Koryak Highlands | South Chukotka |  |
| Kaipilgyn lake, Koryak Highlands | South Chukotka |  |
| Pekulnei lake | South Chukotka |  |
| Third River, Koryak Highland | South Chukotka |  |
| Shlem lake | South Chukotka |  |
| Vaamochka Lake | South Chukotka |  |
| Vaamochka-Pekulnei lake watershed | South Chukotka |  |
| North of Pekulnei lake | South Chukotka |  |
| Mountains in Koryak Highlands | South Chukotka |  |
| Mainitz Lake, Koryak Highland | South Chukotka |  |
| Velikaya river basin | South Chukotka |  |
