# Supplementary material for: Modeling Eastern Russian High Arctic Geese (Anser fabalis, A. albifrons) during moult and brood rearing in the ‘New Digital Arctic’
Source: Sci Rep. 2021 Nov 11;11:22051. doi: 10.1038/s41598-021-01595-7 (PMC8586028; doi:10.1038/s41598-021-01595-7)

Supplement 5. Model Data and Model details

(See also table 2 in the manuscript for model predictor ranks)

*Anser fabalis* brood: top predictor 1-dimensional


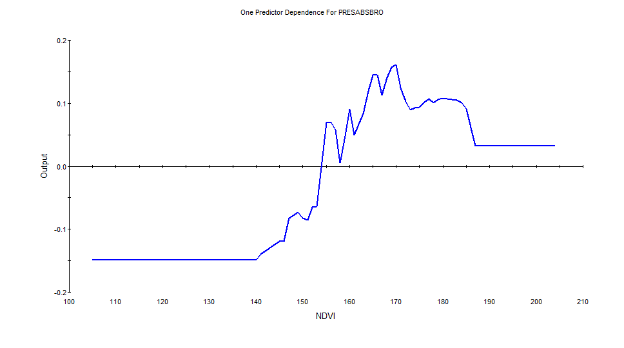


*Anser fabalis* brood: top predictor 2-dimensional


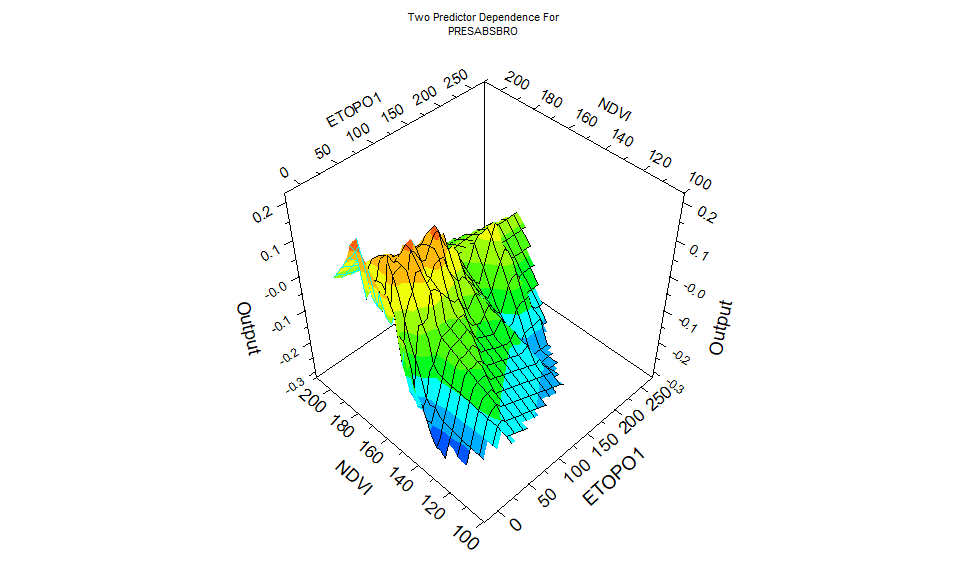


*Anser fabalis* non-breeder : top predictor 1-dimensional


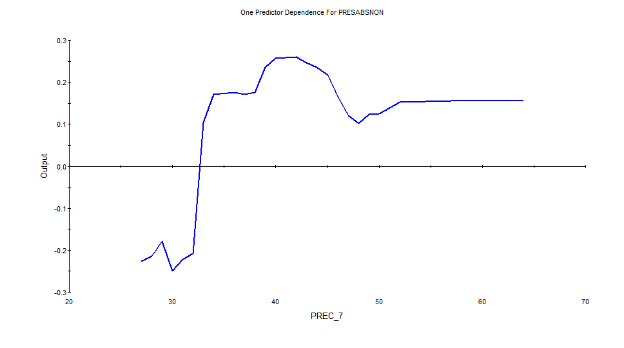


*Anser fabalis* non-breeder: top predictor 2-dimensional

*
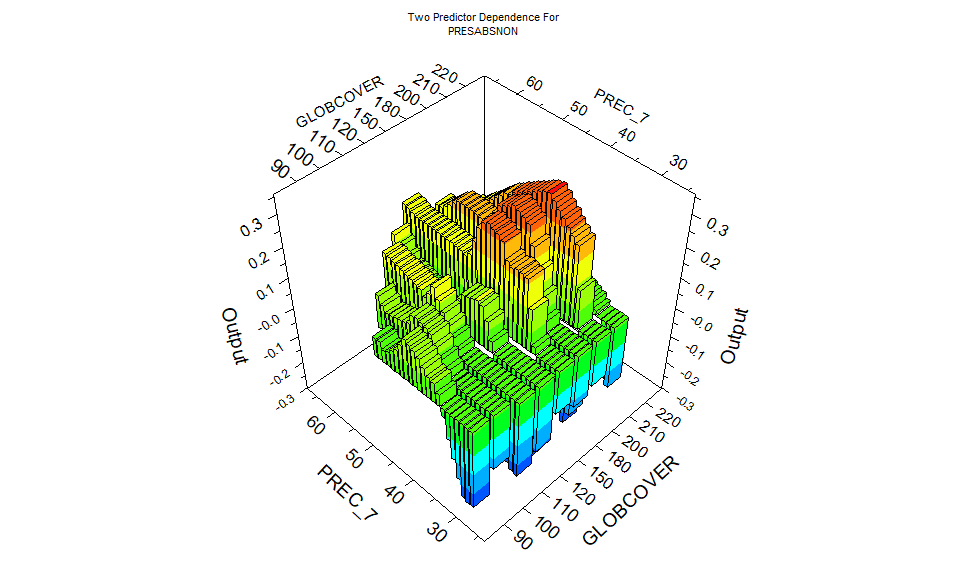
*

*Anser albifrons* brood: top predictor 1-dimensional


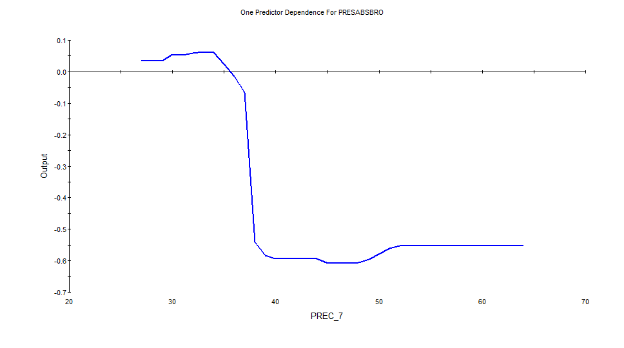


*Anser albifrons* brood: top predictor 2-dimensional


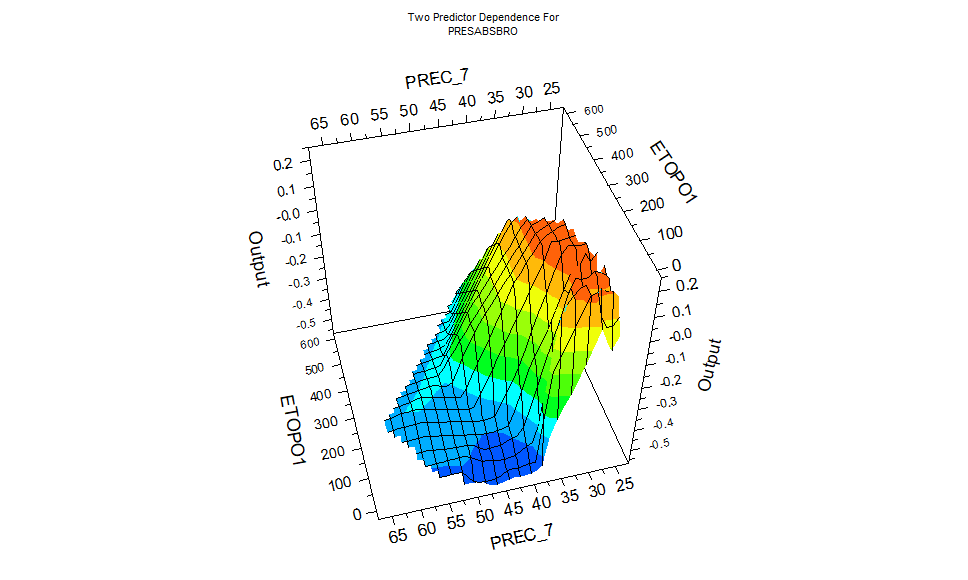


*Anser albifrons* non-breeder : top predictor 1-dimensional


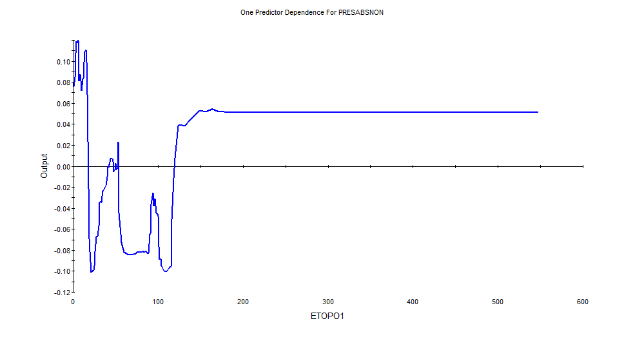


*Anser albifrons* non-breeder: top predictor 2-dimensional


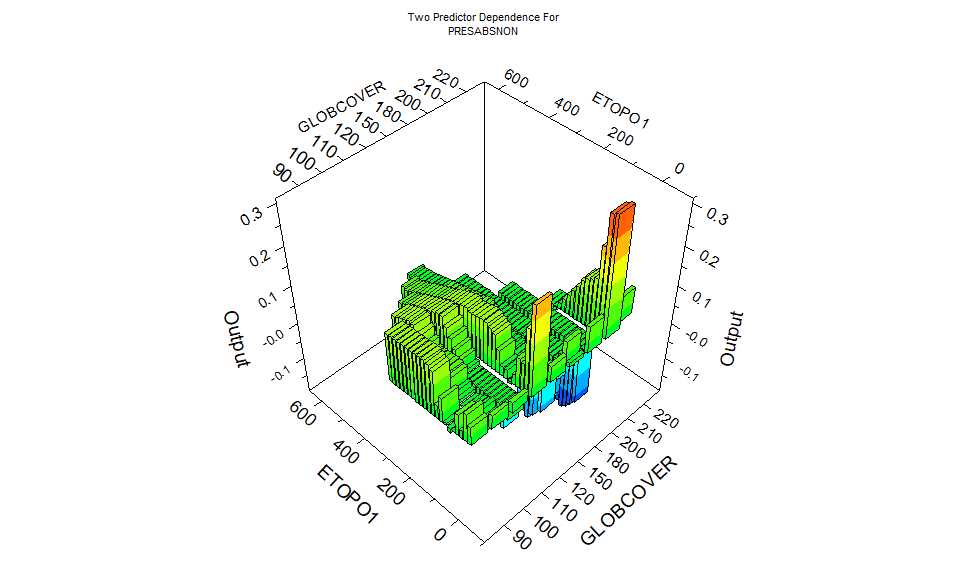

Supplement: Supplementary file 10 — Supplementary Information 10. [file 41598_2021_1595_MOESM10_ESM.docx]
