# Supplementary material for: Modeling Eastern Russian High Arctic Geese (Anser fabalis, A. albifrons) during moult and brood rearing in the ‘New Digital Arctic’
Source: Sci Rep. 2021 Nov 11;11:22051. doi: 10.1038/s41598-021-01595-7 (PMC8586028; doi:10.1038/s41598-021-01595-7)
Supplement: Supplementary file 11 — Supplementary Information 11. [file 41598_2021_1595_MOESM11_ESM.docx]

**Supplement Materials Overview**

Supplement 1: Bird Data with ISO-compliant metadata

GIS shapefiles and associated metadata of tables 1a and 1b

Supplement 2: Six GIS Data layers with details and ISO-compliant metadata

Data layer details and GIS layers (Note: a large tiff file zip archive is currently available from FH due to size restrictions with the journal archive site; contact authors for details)

Supplement 3: List of Survey Location names (following anglicized local geographic names; survey coordinates presented in Supplement 1)

Supplement 4: Model Data: GIS Surface overlays and predictions

Supplement 5: Model Data and Model details
